# Taking guidance from parents involved in a longitudinal birth cohort – the ROLO family advisory committee

**DOI:** 10.1186/s40900-020-00200-x

**Published:** 2020-04-28

**Authors:** N. M. Walsh, E. C. O’Brien, A. A. Geraghty, D. F. Byrne, A. Whelan, S. Reilly, S. Murray, C. Reilly, E. Adams, P. M. Farnan, F. M. McAuliffe

**Affiliations:** UCD Perinatal Research Centre, School of Medicine, University College Dublin, National Maternity Hospital, Dublin, Ireland

**Keywords:** Patient and public involvement, Engagement, PPI, Children, Eating behaviour

## Abstract

**Background:**

The ROLO Study (Randomised cOntrol trial of a Low glycaemic index diet in pregnancy to prevent macrosomia) was a randomised control trial conducted between 2007 and 2011 to examine if a low glycaemic index (GI) diet could reduce the incidence of macrosomia. The ROLO Family Advisory Committee is a self-selected group of parents who are involved in the longitudinal follow-up of the ROLO Study. The committee was established in 2017 and the goal is to achieve a partnership between ROLO families and researchers, leading to improved research quality, relevance, and outcomes. This research method is termed “Public and patient involvement (PPI)” and describes how researchers collaborate and engage with the public in order to make research more relevant to them.

**Methods:**

The ROLO study mothers and children have been prospectively followed-up at multiple time points post-pregnancy. In October 2017, all women were invited to join the ROLO Family Advisory Committee via email or via advertisement on the ROLO Study Facebook page. Fathers and other guardians of the study children were also invited to join. Two annual meetings with the research team and parents were held in 2018 and 2019. The meetings were recorded, transcribed verbatim by researchers, and thematically analysed.

**Results:**

Parents provided opinions on the areas they felt should be explored within the ROLO study using information that was collected up to the current follow-up point. They also shared views on research interests which were of importance to them. These topics included; child mental health, fussy eating in childhood and healthy eating policies in schools. Mothers were much more concerned about factors which influenced their child’s health rather than their own. Incorporating an element of PPI to this study was found to be a positive learning experience for participants and researchers.

**Conclusion:**

The involvement of parents has enriched the research agenda at the UCD Perinatal Research Centre. We will continue to engage with the parents of the ROLO Study and plan to involve the children to explore their opinions at the next opportunity.

## Plain English summary

The ROLO study was carried out between 2007 and 2011 at The National Maternity Hospital Dublin, Ireland. It involved 800 women who were pregnant with their second child and who had previously given birth to an infant weighing over 4 kg. These women either received dietary advice from a dietitian, or no dietary advice to see if a specialised diet would make a difference to the weight of their child. The mothers and children involved in the study have been followed-up five times since birth; at 3 months, 6 months, 2 years, 5 years and nine-eleven years.

Public and patient involvement describes how researchers engage with and involve the public in research to make it more relevant [1]. All parents involved with the ROLO Study were invited to join the ROLO Family Advisory Committee and to attend our annual meetings, with 21 parents expressing an interest in joining. This committee was set up in 2017 and its goal is to build and maintain a strong relationship between ROLO families and researchers. As researchers, it is important for us to understand what is important for parents in terms of research and learn how we can make it more relevant for parents and children.

At the meetings we asked parents their opinions on current projects and what research they would like to see in the future. We also discussed different areas of health and nutrition, especially in relation to children.

As a result of talking to the parents, we found out that mothers cared more about research relating to the children than research relating to them. Parents found the relationship between diet in pregnancy and child health interesting. They also wanted to see research looking at the effect of social media on child mental health and why some children are fussy eaters. All present agreed that all children and teenagers should know how to eat and live healthily. We will use these findings to improve our current studies, as well as plan future research.

## Introduction

The ROLO Study (Randomised cOntrol trial of a Low glycaemic index diet in pregnancy to prevent macrosomia) was a randomised control trial conducted between 2007 and 2011 to examine if a low glycaemic index (GI) diet could reduce the incidence of macrosomia which occurs in 30–50% of second pregnancies [2]. This is of clinical importance as macrosomic infants are predisposed to a number of adverse neonatal and obstetric outcomes, while there is also increased risk of complications during birth for the mother [2].

Longitudinal birth cohorts are the cornerstone of evidence in the field of the developmental origins of health and disease [3]. Follow-up of children and their parents from pregnancy to childhood and beyond enables detailed prospective data collection and complex analysis adjusting for multiple time points and confounders. The benefit of such studies is that researchers can clearly delineate temporal relationships between exposures and outcomes; however, they are not without challenges. Retention of participants in birth cohorts can prove difficult unless strategies to engage participants are carefully considered and employed [4]. One such relatively new method for engagement with participants in research studies is termed Public and Patient Involvement (PPI).

PPI describes the many ways in which researchers interact with those at the centre of the research and for those with whom is it relevant. Participants are kept informed of research relevant to them, and in some cases steer the direction of the research, which often may result in increased participant support and participation due to increased involvement in the research process [1]. This process ensures that the opinions and experiences of participants are taken into account when conducting research. Participants and their families are also given the opportunity to raise any concerns or ask any questions that they may have around the research. This involvement serves to increase the relevance of the research being conducted, as well as being beneficial for both the researchers and the participants. It also maintains a strong and positive relationship between researchers and participants [5].

There are many different options when considering how to involve the public in research. Members of the public can act as joint grant holders or co-applicants on a research project and be involved in identifying research priorities, they can be members of a project advisory or steering group, assist in developing patient information leaflets or other research materials, or undertake interviews with research participants [6]. Although PPI groups are becoming more common within many charities and health organisations, PPI groups among research participants are less common. Feedback from participants involved in PPI with the Irish Cancer Society reports that this involvement was not only beneficial for the researchers in terms of grant proposals but also for the participants, who felt they were contributing to helping cancer patients in the future and expressed the experience as being ‘interesting’ and ‘full of learning’ [7]. Indeed, the Irish Cancer Society prioritises PPI in their research as they are “committed to putting patients, families, survivors, supporters and the public at the very heart of what we do” [8].

The team involved with the ROLO longitudinal birth cohort study [2] established The ROLO Family Advisory Committee in 2017 and has benefited greatly from this partnership. This committee was established in 2017 in order to continue to engage with participants and to maintain follow-up rates. It also recognises the participants on-going support for the study provides them with a platform to voice their opinions on the study. The goal of the ROLO Families Advisory Committee was to achieve a partnership between ROLO families and researchers, leading to improved research quality, relevance, and outcomes. The aim of engaging with parents from the ROLO study was to understand key outcomes of importance relating to their own health and their children’s health, and to apply these insights to future research questions and grant funding proposals. This report aims to outline the process for establishing the PPI group and detail the positive outcomes that both researchers and members of the committee have gained. This includes the benefits of PPI in terms of contributing to research, informing both researchers and participants and potentially learning more about scientific research. This report will also outline the thoughts and opinions of the parent participants regarding the ROLO Study and key research areas of importance.

For the purpose of this paper, participants are defined as those who are part of the ROLO Study, i.e. the 800 pregnant women who were recruited initially and the child they gave birth to. As much of the interaction researchers had with participants was with the mothers, fathers and guardians of the children, much of this paper refers to the input of the parents rather than the participants. The term “involvement” will incorporate being part of the conduction and dissemination of research.

## Methods

### The ROLO study

As previously mentioned, the ROLO study (Randomised cOntrol trial of a LOw glycaemic index diet in pregnancy to prevent macrosomia) was a randomised controlled trial conducted between 2007 and 2011, at The National Maternity Hospital (NMH), Dublin, Ireland. In brief, 800 secundigravida women who had previously given birth to a macrosomic infant (> 4000 g) were randomised to receive either low-glycaemic index (GI) dietary advice from the research dietitian, or standard usual care (no dietary advice) with the aim to reduce recurrence of macrosomia [2].

**Fig. 1:**
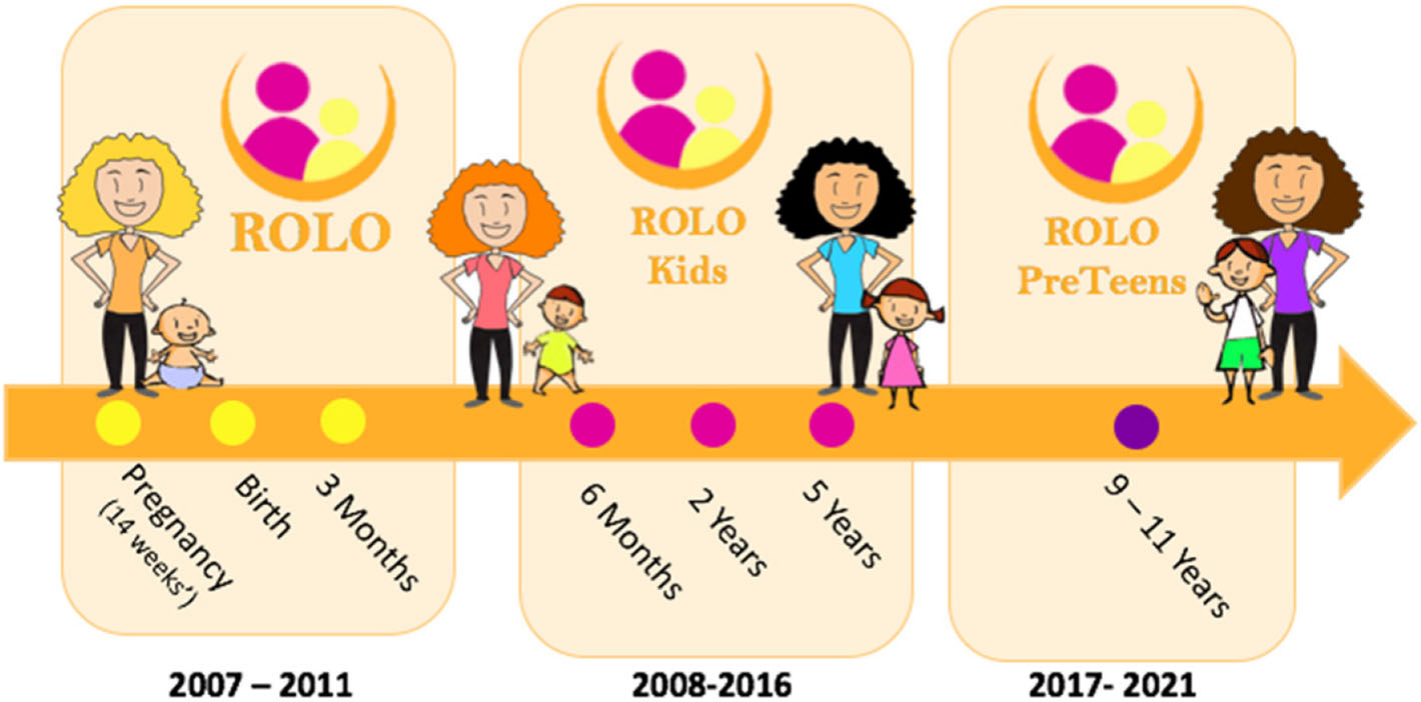
Infographic of the ROLO longitudinal follow-up study and the time points

The ROLO study mothers and offspring have been prospectively followed-up at multiple time points; 3 months, 6 months, 2 years, and 5 years post-intervention. The 9–11 year follow-up is ongoing (see Fig. 1).

### Setting up of the ROLO family advisory committee

In October 2017, all mothers still involved in the longitudinal study were invited via email or the ROLO Study Facebook page to join the ROLO Family Advisory Committee. The email and Facebook post included a brief summary of the aims of the ROLO Family Advisory Committee and what it entailed such as voicing their views on what questions should be explored in terms of the ROLO Study, being involved in identifying research priorities and providing their input on grant proposals for research funding. Fathers and any other guardians or parents of the study children were also invited to join. Initially, 16 participants signed up to be part of the committee, but there is an ever-increasing interest in being involved. In total, 19 mothers and two fathers are now members of the ROLO Family Advisory Committee. Out of the 16 participants who originally signed up, eight were randomised to the control group of the study and eight were in the intervention group.

### Interaction with participants via Facebook

As part of the participant communication and study retention strategies [4] the ROLO study has a dedicated Facebook page. The ROLO Study Facebook page is a closed, private “friend” page. The research team have control over who can add the ROLO Study as a “friend”, thus ensuring that only participants are part of the group. In November 2017 a short online survey was put up on the ROLO Study Facebook page to ask mothers their opinions about their current health and how pregnancy can be a predictor of future health. Mothers were asked to rate their level of perceived importance (on a 4-point Likert scale) relating to weight gain during pregnancy, feeding patterns in early life, predictive blood tests for future health, and lifestyle choices and pregnancy factors that can affect child health. Mothers were encouraged to ask their child if they were interested in the effect of diet and exercise on health. This online survey was conducted in order to gauge the areas of most interest to parents in terms of their child’s health. This was considered important in order to ensure that their research interests were being taken into account when analysing data from the ROLO Study.

### Annual round-table meetings of the ROLO family advisory committee

In January 2018 and February 2019, parents who had expressed interest in joining the committee (*n* = 21) were contacted by the research team to invite them to attend the first and second ROLO Families Advisory Committee meeting, which took place in the National Maternity Hospital. The main aim of these meetings was to provide parents with updates on the research being carried out and gather their feedback and opinions regarding on-going research.

The topics discussed at these meetings included macrosomia, the low GI intervention and the dietary advice received by mothers participating in the ROLO study, the study randomisation process, and how participants felt about being in the control group. Parents also provided opinions relating to practical aspects of the follow-up appointments. The committee was updated on results coming from the ROLO study to date and given an insight into the next stage of the ROLO study, including grant and funding applications. Parents were asked a range of questions on pre-discussed topics such as research areas they would like to know more about, ideas for future research, suggestions on how to improve the research, and to voice any concerns they may have had. Other ongoing studies at the centre were outlined and feedback was requested as to how these studies could be improved from a participant perspective. Parents were also asked if they would be interested in reading this report on its completion.

The duration of the meetings was approximately 2 hours and refreshments were provided. Participants did not receive an incentive for attending. Written consent was obtained from each committee member to record the meetings. A Sony digital audio device model UX560 was used for this purpose. At the first meeting in 2018, the researchers (EOB, AG, DB, and FMcA) met with five mothers, and at the second meeting in 2019 the researchers (EOB, AG, NW, and FMcA) met with three mothers and one father. The discussion with the parent committee members focused on what areas they would like to know more about and their concerns relating to children’s health. The audio recordings were transcribed verbatim and themes were identified by a member of the research team by analysing the minutes of the meeting. A basic content analysis was conducted by reading through the transcription of the meeting and we conducted a tabulation to summarise the points which were discussed the at meeting (Table 1). Themes were characterised by their frequency. As the numbers at the meeting were small, a lot of areas of interest over-lapped and subthemes were identified within initial themes during discussions. For example; when discussing the theme of body image, subthemes such as mental health, sports participation and the role of social media on nutritional health were identified (as seen in Table 1). Although some of these themes overlap slightly, it was important to clearly identify all those with high prevalence in the discussion as this signifies their perceived importance among parents.

**Table 1:** Key Themes and Subthemes Identified at the ROLO Family Advisory Committee Meetings

Themes	Subthemes	Description
Markers of Health	N/A	The idea of a general blood test in pregnancy that could predict health risks for children in later life was viewed positively
Fussy Eating	N/A	High level of interest in this topic as it can affect meals. It was also suggested to look at differences between siblings when it comes to food-choice and preferences.
Mental Health	• Wellbeing & Behaviour	It was felt that body image influenced the high drop-out rates from sport seen among teenage girls. Highlighted the link between body image, social media and sports participation.
	• Body Image	
	• Sports Participation	Particular interest in the area of wellbeing and behaviour, especially the societal influence of social media and conforming to “norms” for children. Interest in examining if there was a relationship between a woman’s diet and mood during pregnancy and her mental health ten years on.
	• Influence of social media	
Public Health Nutrition	• Dietary Advice	Reported a lack of clarity surrounding the need for supplements, vitamin D in particular. Highlighted the need for more information on supplementation for the general public.
	• Micronutrient Supplementation	
	• Healthy Eating Policies in Schools	Peers can have a strong influence on the foods a child will choose to consume, school-based initiatives such as ‘Food Dudes’ made fruit and vegetables more acceptable in school lunches.
	• Making Healthy Food Choices	
		Parent committee members would like to see a food pyramid aimed specifically at children and younger teenagers.

## Results

A strong partnership between ROLO Study participants and researchers was developed through the implementation of elements of PPI. This included regular updates on findings from the ROLO Study via Facebook and emails, polls conducted on Facebook and the establishment of the ROLO Family Advisory Committee. Participants were encouraged to provide their feedback on all aspects of the ROLO Study which in turn enabled researchers to identify areas of the ROLO Study to improve on. For example, researchers identified that most participants preferred to be contacted via email and that facilitating study days on the weekends suited the majority of participants. As a result of increased contact between participants and researcher, a strong relationship was formed which we envisage will maintain follow-up rates as participants felt more involved in decision making and the identification of research priorities.

The discussion with the parent committee members allowed for key areas of importance to parents to be identified. Hence, researchers were alerted to topics which may not have been previously considered for further research. These areas focused in on their concerns relating to their children’s health and areas they would like to know more about. Key themes were those which were discussed at length and those which parents felt most passionate about. Parents were encouraged to voice their opinions with as few interruptions as possible from researchers in order to allow for the natural development of subthemes within discussions. Table 1 provides brief information about the key themes and subthemes that were identified. Topics which generated the most discussion included fussy eating and the influence of society and social media on children in terms of mental health, wellbeing and sports participation. There was also significant discussion around where children obtain their nutritional information and the role healthy eating policies at school have in this. Parents suggested creating a survey to be completed by children at the pre-teen phase of the study to gauge the impact of societal ideals on children and its impact on their mental health. Parents indicated that they found the relationship between diet in pregnancy and child health interesting. They also highlighted that they cared more about research relating to the health of their child rather than research relating to them; however, they did suggest that it would be interesting to see if a woman’s diet in pregnancy was associated with her mental health 10 years on. The idea of a blood test in pregnancy that could predict health risks for children in later life was viewed positively and welcomed by the family participants. It was also felt that dietary advice for mothers should encompass the family not just mother and to factor in time constraints when providing this advice. Mothers also felt that rather than outcomes of studies focusing on weight gain, results should focus on health outcomes.

The introduction of the PPI aspect to the ROLO Study was found to be beneficial to both the parents and the researchers. Parents felt as though they were “giving back” to research while also improving on their scientific knowledge. In addition to attending annual meetings, parents were also involved in grant application, contributed to presentations at conferences and assisted with reviewing of manuscripts. Researchers involved in this body of work gained an insight into the research areas of importance for parents and received positive feedback on the study thus far.

## Discussion

### Topics of interest to parents

Although the ROLO study focuses on both mothers and children, from the PPI meetings it was evident that parents had greater concern for outcomes relating to their children’s health, rather than their own. Interest focused on topics that could influence the health of the children both now and later in life. These topics ranged from potential predictors of health risks, dietary habits and information, contributing factors to health and study outcomes.

One of the areas discussed was the issue of child fussy eating at home and how it affected mealtimes. Parents felt that this was an area which should be investigated and one which they would be very interested in. It was also suggested that looking at differences between siblings in terms of food preferences could be beneficial. Research indicates that almost half of parents report that their child is a fussy eater at some stage in their first 6 years of life [9]. It was interesting that this PPI meeting reflected concerns among parents in the wider population. A recent study reported that 53.9% of parents had concerns about child fussy eating but listed lack of time and tantrums as barriers to address these concerns [10]. In previous parent focus groups conducted in studies of this nature, parental concern regarding their child’s eating habits is usually the dominant topic of the discussion and the general opinion is that a fussy eater can make meal-times more stressful. Additionally, there are concerns that fussy eating in childhood could affect them in the future, potentially due to reduced food choice and preferences or the belief that they may be lacking nutrients [11]. There was a particular interest in the area of wellbeing and behaviour, especially the societal influence of social media and conforming to “norms”. On this topic, it was felt that asking children taking part in the pre-teen phase of the study to fill out a survey to gauge their opinions would be interesting and “could be a real eye opener”.

Another recurring topic was the area of body image and mental health. It was felt that body image also influenced the high drop-out rates from sport seen among teenage girls [12] and that there needs to be a system in place to support girls and encourage them to continue in sport. It was suggested to link body image, social media, and sports participation, to examine childrens’ opinions on these topics in the next phase of the study. Mental health in childhood is a huge issue in today’s society [13] and it was thought that examining this topic could provide scope as to how mental health in children and teenagers is affected and what could be done to help this problem. It was consistently highlighted that children and teenagers should know what was healthy and what could be done to support health – nutritionally, physically and mentally. Parent committee members also felt it would be interesting to see if there was a relationship between a woman’s diet and mood during pregnancy and her mental health 10 years on.

There was a discussion about micronutrient supplements amongst parents and researchers. Parents also voiced their concerns about the lack of clarity surrounding the need for supplements, vitamin D in particular. They highlighted the need for more information on supplementation for the general public as they felt it was quite ambiguous, and for medical professionals to also be aware of supplementation uses and needs.

Healthy eating policies in schools were also discussed as parents remarked how peers can have a strong influence on the foods that a child will choose to consume. While it was acknowledged that initiatives such as ‘Food Dudes’ [14] made fruit and vegetables more acceptable in school lunches, it was accepted that often school lunches cannot be policed by teachers to ensure they are healthy. However, as teachers have such an influence on children, it is vital that they enforce healthy messages and are important sources of information for children. It is, of course, imperative that these messages are correct and evidence based. Following on from this, there was a suggestion to look at the influence of school on eating behaviours. Parents would also like to see a food pyramid aimed specifically at children and younger teenagers which focuses on colour and encourages children to make their own healthy food choices.

The fact that parents were positive about the use of a blood test in childhood to predict health outcomes should be considered by researchers for future studies as parents viewed it as relevant. It is also of note that parents recognised the importance of blood tests in determining health status and understood that this method was more useful that other methods of determining health status.

We predict that the identification of these themes will lead to an increase in the relevance of research outcomes. Researchers can focus their work on areas which will be of most interest to parents of children of all ages and which may not have been studied before. For instance, fussy eating in childhood was pinpointed as a topic which concerned parents and subsequently a grant was secured to facilitate research in this area.

### Role and benefits of PPI

From the point of view of a researcher, the methods of PPI which were incorporated into the ROLO Study (increased contact and feedback, surveys, establishment of a committee, participant contributions to manuscripts, conferences and grant proposals) have suited this study and have provided a platform for participants to increase their involvement in research and improves the relevance of the ROLO Study. When analysing the attendance at meetings, it appears it may be necessary to conduct meetings at other stages during to year to further increase participant involvement. This may involve facilitating meetings at weekends or in the evenings or may involve the use of video technology to allow participants to “dial in” if they are not in a position to attend in person.

PPI can greatly enrich research and be of benefit to both participants and researchers. PPI has had very positive feedback from other studies reporting that participants gained new knowledge and skills while growing self-confidence [7]. At the ROLO Family Advisory Committee parent members reported feeling that they were obtaining useful information about their children while also being able to contribute to research. They were in favour of children being involved in meetings and also having a say into what research was conducted. The parents agreed that the older sibling could also be involved in ROLO research going forward, emphasising the importance of a family approach for studies of this nature. This highlights the positive influence PPI can have on parents and their attitudes towards research. The use of PPI for the ROLO study is important as the study changes as the children get older so study ideas and methods must be tailored to best suit the study participants. With the involvement of the parents the direction of the research and the agenda can be altered ultimately leading to much stronger and relevant research being conducted. Other studies which have involved the public or participants have also reported that this collaboration assisted in making reports more hard-hitting, accessible, and useful to the target audience [1]. Specifically, for the research team, PPI has many benefits in terms of developing and improving understanding of a condition or concerns of the participants [1]. In addition to assisting research by identifying areas of importance, PPI encourages involvement in subsequent research [15]. Undoubtedly PPI can be hugely valuable at every stage of the research process by helping to ensure that funding is appropriately prioritised, that research evidence is relevant to participants, by improving recruitment and retention rates, and supporting the uptake of research in practice [16].

### Future research

At the meeting the future direction of the ROLO study was discussed and it was suggested that a children advisory committee could be set up with just the children and the research team present to obtain information solely from the child’s perspective. We are currently in the process of identifying the most effective way of interacting with the child participants and what topics we feel would be of benefit to discuss with them. A topic which was suggested to focus on for the teenage follow-up stage was sources of information on nutrition and exercise. It was also suggested to investigate an app for teenagers which they could use to obtain activity-specific food ideas and recipes to help them better understand nutrition and assist them in following a healthy lifestyle. This app would provide teenagers with reliable nutritional information and deter them from looking for nutritional advice on social media. Research into the area of sexual health was also encouraged as the children involved in the ROLO Study are approaching puberty but there was little further discussion around this topic. In terms of future research, examining relationships between a mother and child’s blood markers of health was better received by the parents than looking at the relationship between measures such as blood pressure levels. Yet parents also felt that if these relationships are beneficial for clinicians to examine then they should be looked at. Sleep patterns in children was supported as an important topic to look at and it was emphasised again the potential benefit of including other siblings from these families for comparisons.

We are constantly looking to increase to number of members on the ROLO Family Advisory Committee and will continue to do so by interacting with all participants regularly and informing them about the role and benefits of the committee. This paper focuses on families’ thoughts and opinions and is not an in-depth analysis of their experience. Hence, in the future we aim to conduct a further piece of research to explore the ROLO participants’ experiences of being involved in research.

### Strengths and limitations

All parent members in the ROLO Advisory Committee are a self-selected group that participate in the ROLO Study and assist in improving the strength of research being conducted. Meetings were recorded and transcribed in order to ensure all topics of interest were correctly identified and all participants had sufficient time to express opinions and views in a relaxed environment. As there was a small group of parents in attendance at the meetings, it has to be considered that their opinions may not necessarily represent all of the parents involved in the ROLO study. Meetings were held during the week which may have been difficult for parents who are working or those with young children to attend. It is vital to consider hard to reach groups when encouraging attendance at these meetings. In order to reach as many groups as possible, all channels of communication were utilised. These included social media platforms (Facebook), email, letters, phone calls and text messages. An email of invitation to join the ROLO Family Advisory Committee is sent to all participants prior to any meetings of the committee highlighting the benefits of PPI and the role of the committee. This allows for the consistent growth of the committee and encourages those who may not have been interested at the time to consider becoming part of the committee. Regular reminders about meetings are also sent via email and text message.

## Conclusion

As a result of the ROLO Family Advisory Committee and the PPI component of the ROLO Study, key areas have been identified to prioritise, leading to a more focused and stronger study, with researchers obtaining a clearer view of what’s important to parents. Taking the parents’ suggestion of examining “fussy eating”, we secured a grant to conduct research in this area. This would not have been achieved without the input from the parents. The ROLO study research agenda has certainly been enriched by PPI and we encourage greater involvement of patients and participants in research going forward in this important area of inter-generational health.

## Supplementary information

**Additional file 1.** GRIPP2 Short Form.

## Data Availability

The datasets used and/or analysed during the current study are available from the corresponding author on reasonable request.

## Abbreviations

PP: Public and Patient Involvement
ROLO: Randomised cOntrol trial of a LOw glycaemic index diet in pregnancy to prevent macrosomia
NMH: National Maternity Hospital
