# Supplementary material for: Taking guidance from parents involved in a longitudinal birth cohort – the ROLO family advisory committee
Source: Res Involv Engagem. 2020 Apr 28;6:16. doi: 10.1186/s40900-020-00200-x (PMC7189585; doi:10.1186/s40900-020-00200-x)
Supplement: Supplementary file 1 — Additional file 1. GRIPP2 Short Form. [file 40900_2020_200_MOESM1_ESM.docx]

**GRIPP2 short form**

| **Section and topic** | **Item** | **Reported on page No** |
| --- | --- | --- |
| 1: Aim | Report the aim of PPI in the study | 4 |
| 2: Methods | Provide a clear description of the methods used for PPI in the study | 4,5,6,7 |
| 3: Study results | Outcomes—Report the results of PPI in the study, including both positive and negative outcomes | 7,8 |
| 4: Discussion and conclusions | Outcomes—Comment on the extent to which PPI influenced the study overall. Describe positive and negative effects | 8,9,10,11,12 |
| 5: Reflections/critical perspective | Comment critically on the study, reflecting on the things that went well and those that did not, so others can learn from this experience | 10,11,12 |
